# Case report: atrial septostomy as a bridge to lung transplantation in a patient with venovenous extracorporeal membrane oxygenation

**DOI:** 10.1097/MD.0000000000028889

**Published:** 2022-02-18

**Authors:** Jiwon Ryoo, Jung Huh, Hee Sun Cho, Jin-Jin Kim, Seok Chan Kim, Jongmin Lee

**Affiliations:** a Division of Pulmonary and Critical Care Medicine, Department of Internal Medicine, College of Medicine, The Catholic University of Korea, Seoul, Republic of Korea; b Department of Internal Medicine, Seoul St. Mary's Hospital, College of Medicine, The Catholic University of Korea, Seoul, Republic of Korea; c Division of Cardiology, Department of Internal Medicine, Seoul St. Mary's Hospital, College of Medicine, The Catholic University of Korea, Seoul, Republic of Korea.

**Keywords:** atrial septostomy, extracorporeal membrane oxygenation, lung transplantation

## Abstract

**Introduction:**

Advances in critical care management have led to the recent increase in the use of extracorporeal membrane oxygenation (ECMO) as a bridge to lung transplantation (LT). Patients with respiratory failure requiring venovenous ECMO usually experience progressive right ventricular (RV) failure. Diagnosis and treatment of RV failure during ECMO are essential for improving the prognosis of patients.

**Patient concerns:**

A 28-year-old female patient underwent allogeneic hematopoietic stem cell transplantation (HSCT) from a matched unrelated donor for acute myeloid leukemia presenting with progressive dyspnea.

**Diagnoses:**

Computed tomography revealed multifocal patchy peribronchial and subpleural ground-glass opacities in both lungs, and the patient was clinically diagnosed with cryptogenic organizing pneumonia.

**Interventions and outcomes:**

Despite intensifying systemic corticosteroid therapy, her symptoms deteriorated, and mechanical ventilation and ECMO were applied. During treatment, her respiratory failure continued to progress, and systemic hypotension developed. An echocardiogram showed evidence of RV failure, and percutaneous atrial septostomy was performed for RV decompression. After a balloon atrial septostomy was performed, RV failure of the patient improved, and LT was successfully performed.

**Lessons:**

We report the first case of atrial septostomy as a successful bridge to LT in a HSCT recipient with venovenous ECMO. Atrial septostomy could be an option for management of RV failure during ECMO. Further studies need to be conducted to validate the effect of atrial septostomy in patients with RV failure during ECMO.

## 1 Introduction

Hematopoietic stem cell transplantation (HSCT) is an important treatment for hematologic malignancies. Pulmonary dysfunction is a significant complication following allogeneic HSCT and is associated with morbidity and mortality.^[1]^ Noninfectious pulmonary complications of allogeneic HSCT include cryptogenic organizing pneumonia (COP) (previously called bronchiolitis obliterans organizing pneumonia) and bronchiolitis obliterans syndrome (BOS). There is an increasing number of lung transplantations (LTs) in selected patients with BOS or COP following HSCT.

Once patients require mechanical ventilation or extracorporeal membrane oxygenation (ECMO), they commonly experience progressive right ventricular (RV) failure.^[2]^ Although there are several strategies to bridge these patients to transplantation, most of them are invasive surgical methods that might cause bleeding or infectious complication. Percutaneous atrial septostomy could allow right-to-left shunting, thus increasing systemic cardiac output, resulting in an increase in systemic oxygen transport without surgical complications.^[3]^ Although a previous study reported the effect of atrial septal defect (ASD) creation as a bridge to LT in patients with primary pulmonary hypertension,^[4]^ to the best of our knowledge, there are no published reports of percutaneous ASD creation in patients with venovenous ECMO awaiting LT. We report the first case of successful percutaneous atrial septostomy for RV failure as a bridge to LT in a patient with venovenous ECMO.

## 2 Case presentation

**Figure 1:**
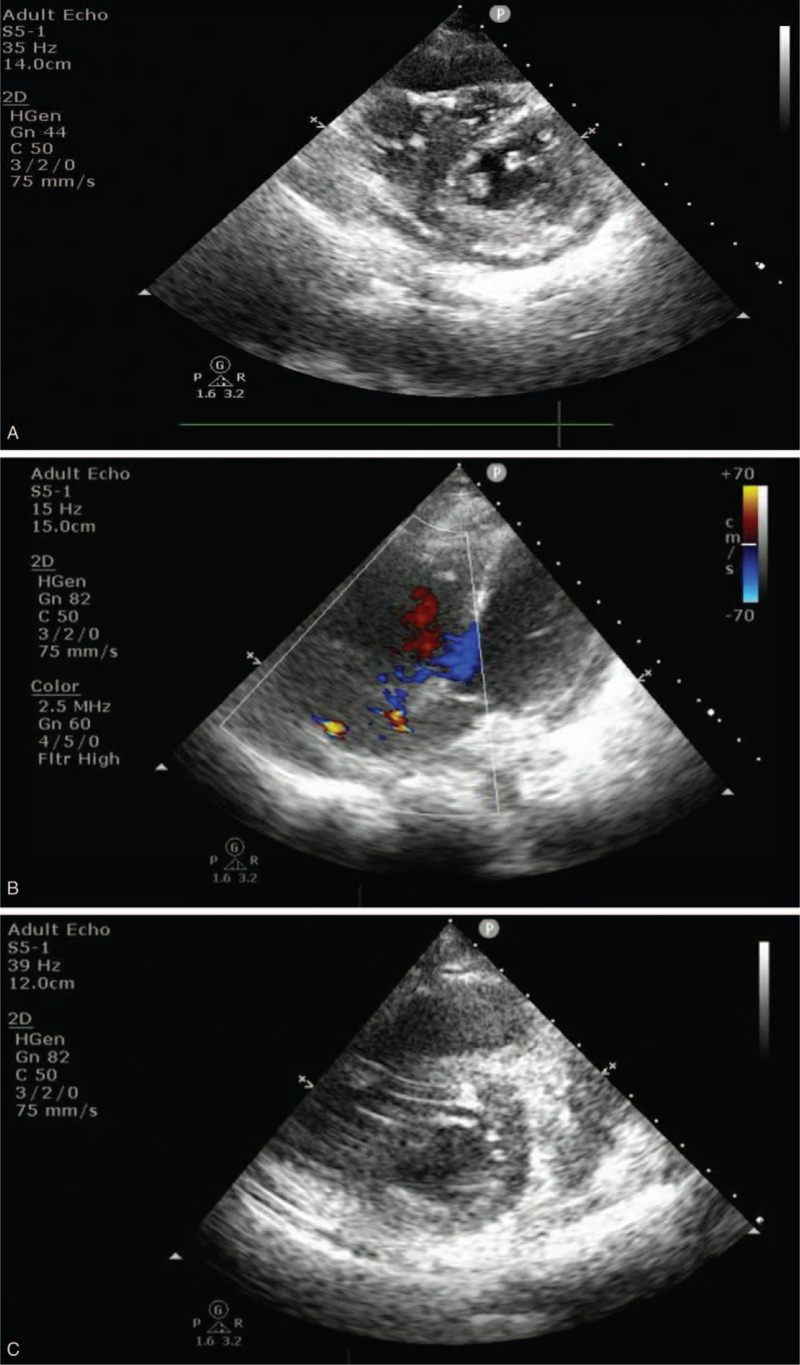
TTE showed an enlarged right ventricle and flattened inverted septum with diminished right ventricular function (A). After a balloon atrial septostomy performed, TTE showed the presence of ASD (B) and the improvement of RV function (C).

**Figure 2:**
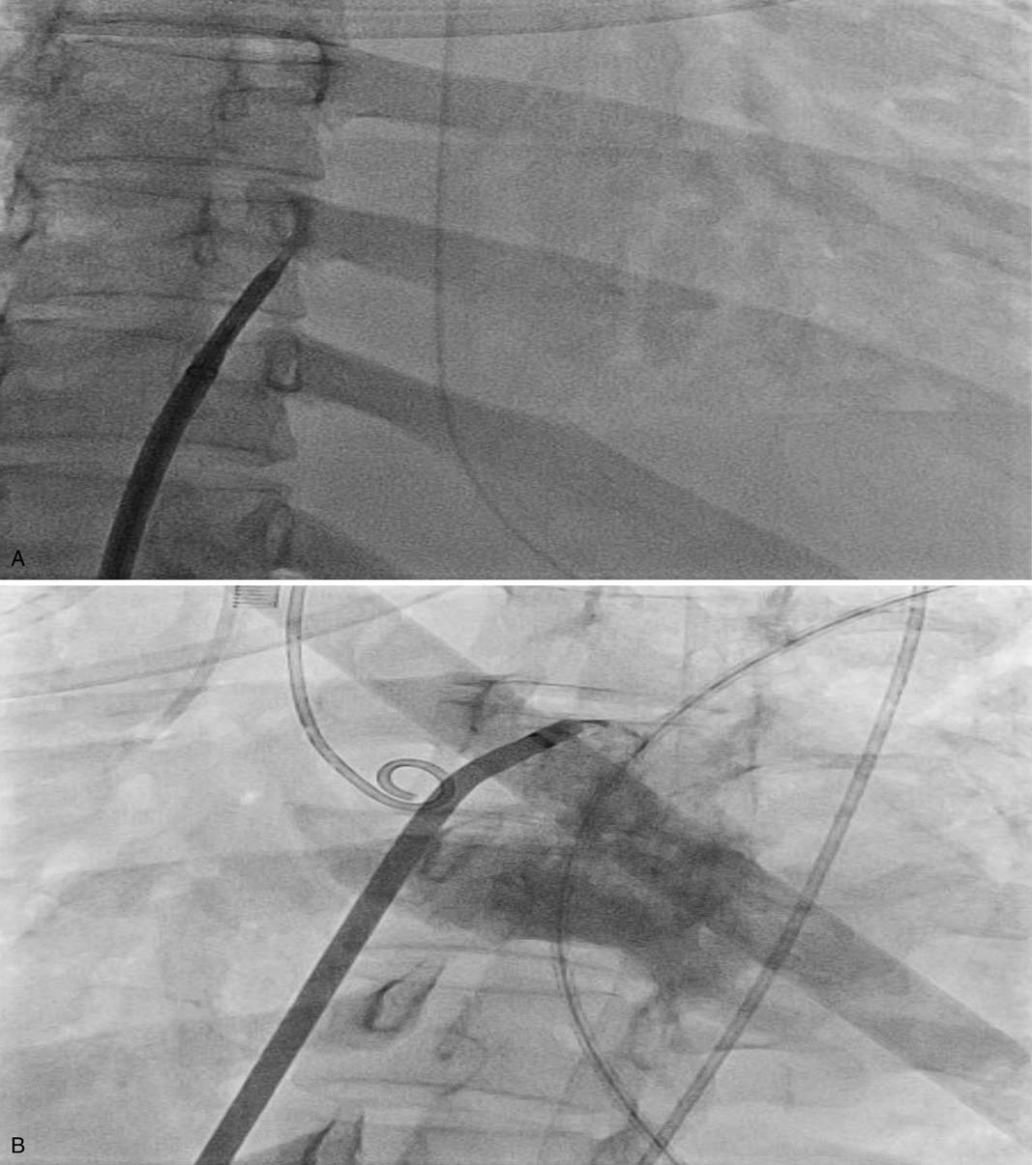
Creation of atrial septostomy by puncture and balloon dilation (A). The presence of septostomy was confirmed by contrast media injection (B).

A 28-year-old female patient underwent allogeneic HSCT from a matched unrelated donor for acute myeloid leukemia. Her post-transplant course was complicated by chronic graft-versus-host disease of the skin and liver. Five months after HSCT, the patient developed progressive dyspnea and facial edema. The patient was admitted to the ward, and work-up for the infectious disease process was negative. An initial transthoracic echocardiogram (TTE) showed normal left ventricular systolic function, and there was no evidence of RV dysfunction. Pulmonary function tests could not be performed because of severe dyspnea. Chest computed tomography showed multifocal patchy peribronchial and subpleural ground-glass opacities in both lungs, and the patient was clinically diagnosed with COP. Despite intensifying systemic corticosteroid therapy, her symptoms deteriorated. On hospital day 9, the patient required invasive mechanical ventilation, and venovenous ECMO was instituted on hospital day 16. Despite further mechanical ventilation and ECMO, her respiratory failure continued to progress, and systemic hypotension developed on hospital day 23. A TTE demonstrated an enlarged right ventricle and flattened inverted septum with diminished RV function (Fig. 1A). Despite meticulous adjustment for mechanical ventilation and volume status, RV failure progressed. Given the contribution of progressive RV failure and hypotension, a decision was made to perform an atrial septostomy. On hospital day 24, a balloon atrial septostomy was performed (Fig. 2). A post-cannulation TTE showed the presence of ASD and the improvement of RV function (Fig. 1B, C), and the patient could be weaned from all inotropes. On post-cannulation day 7, the patient underwent successful double LT, and closure of the ASD was performed simultaneously. Pathological examination of the pneumonectomy specimen was compatible with COP. On postoperative day 2, the patient was successfully weaned off ECMO, and no bleeding or primary graft dysfunction was observed within the first 72 hours. The patient was transferred to the general ward on postoperative day 27 and liberated from mechanical ventilation 7 days later. Follow-up echocardiography demonstrated decreased RV chamber size and improved RV systolic function. At 2 months post-transplantation, the patient was discharged home without complications.

## 3 Discussion and conclusions

We report the first case of atrial septostomy as a successful bridge to LT in an HSCT recipient with venovenous ECMO. Pulmonary complications are one of the major causes of morbidity and mortality in HSCT recipients. When pulmonary complications deteriorate despite medical therapy or mechanical ventilation, LT is the only treatment option. Recently, due to advances in critical care management, the use of ECMO as a bridge to LT has steadily increased.^[^5^,^^6^^]^ However, bridging LT with ECMO has been associated with treatment-related complications.^[7]^

RV failure is a frequent complication of acute respiratory failure.^[7]^ Changes in pulmonary vascular resistance in acute respiratory distress syndrome (ARDS) and the effects of mechanical ventilation may affect RV function.^[8]^ Hypoxic pulmonary vasoconstriction due to severe respiratory failure can contribute to increased RV afterload.^[9]^ Furthermore, lung overdistension due to mechanical ventilation could result in an increased RV afterload. Mechanical ventilation may also influence RV preload; high tidal volume or inappropriate high positive end-expiratory pressure levels might result in diminished venous return and RV preload.^[10]^ Although the incidence of RV failure related to ARDS is controversial, a previous study reported that RV failure was present in more than half of severe ARDS patients with venovenous ECMO.^[2]^ RV failure results in low cardiac output and systemic venous congestion and is associated with poor outcomes in patients with ARDS.^[^11^–^13^]^

Although venovenous ECMO usually improves RV failure in patients with ARDS, in this case, RV failure in the patient progressed during ECMO. There are several causes of progressive RV failure in patients with ARDS and ECMO. Non-pulsatile ECMO flow may sustain RV overload, which can induce a decrease in RV recoil function. Fluid overload can exacerbate RV failure. Moreover, derecruitment or atelectasis after initiating ECMO can increase RV afterload, which can lead to RV failure.^[14]^

When RV failure progresses even after adequately addressing the reversible cause, other options should be considered. Although there have been no previous studies evaluating the effect of atrial septostomy on patients with RV failure during ECMO, we performed atrial septostomy to decompress the right heart as a bridge to LT. Theoretically, an acquired ASD can increase cardiac output and improve systemic oxygen transport. Previous studies have shown that atrial septostomy was successfully performed in LT candidates with isolated RV failure, and several studies have shown improved oxygen transport and hemodynamics up to several months after the procedure.^[^4^,^^15^^]^ A previous animal study showed that an acquired intracardiac shunt combined with venovenous ECMO improved cardiac output and RV pressures with excellent oxygenation and ventilation in an RV failure sheep model.^[3]^ Although atrial septostomy could increase the risk of stroke that could previously be filtered by the pulmonary vasculature, no such side effects were observed in this case. Other potential options for patients with RV failure during venovenous ECMO include inserting an intra-aortic balloon pump or RV assist device, centrally cannulated venovenous ECMO, and switching from venovenous to venoarterial ECMO.^[^8^,^^16^^]^ However, as the patient required systemic anticoagulation, treatment with sternotomy or arterial access was an option we wanted to avoid.

In summary, RV failure could develop in patients with respiratory failure with venovenous ECMO, and atrial septostomy could be an optimal treatment option to decompress the right heart as a bridge to LT. This report could provide another option for respiratory failure patients with RV failure during venovenous ECMO. Further studies are needed to validate the effect of atrial septostomy in patients with RV failure during ECMO.

## Author contributions

JR, JL, and SCK were responsible for patient management and drafted the manuscript. JH and HSJ were the attending physicians throughout the disease course. J-JK performed the percutaneous atrial septostomy and analyzed the result of TTE. SCK supervised this manuscript. All authors have read and approved the final manuscript.

**Data curation:** Jung Huh, Hee Sun Cho.

**Methodology:** Jin-Jin Kim.

**Supervision:** Seok Chan Kim, Jongmin Lee.

**Writing – original draft:** JIWON RYOO.

**Writing – review & editing:** Jongmin Lee.
